# Pseudocarcinomatous hyperplasia of the fallopian tube mimicking tubal cancer: a radiological and pathological diagnostic challenge

**DOI:** 10.1186/s13048-016-0288-x

**Published:** 2016-11-14

**Authors:** Nam Kyung Lee, Kyung Un Choi, Ga Jin Han, Byung Su Kwon, Yong Jung Song, Dong Soo Suh, Ki Hyung Kim

**Affiliations:** 1 Department of Radiology, Pusan National University School of Medicine, and Biomedical Research Institute, Busan, 49241 South Korea; 2 Biomedical Research Institute and Pusan Cancer Center, Pusan National University Hospital, 179, Gudeok-Ro, Seo-Gu, Busan, 49241 South Korea; 3 Department of Pathology, Pusan National University School of Medicine, and Biomedical Research Institute, Busan, 49241 South Korea; 4 Department of Obstetrics and Gynecology, Pusan National University School of Medicine, 179, Gudeok-Ro, Seo-Gu, Busan, 49241 South Korea

**Keywords:** Pseudocarcinomatous hyperplasia of the fallopian tube, Tubal cancer, Pelvic mass

## Abstract

**Background:**

Pseudocarcinomatous hyperplasia of the fallopian tube is a rare, benign disease characterized by florid epithelial hyperplasia.

**Case presentation:**

The authors present the history and details of a 22-year-old woman with bilateral pelvic masses and a highly elevated serum CA-125 level (1,056 U/ml). Ultrasonography and magnetic resonance imaging (MRI) of the pelvis showed bilateral adnexal complex cystic masses with a fusiform or sausage-like shape. Contrast-enhanced fat-suppressed T1-weighted images showed enhancement of papillary projections of the right adnexal mass and enhancement of an irregular thick wall on the left adnexal mass, suggestive of tubal cancer. Based on MRI and laboratory findings, laparotomy was performed under a putative preoperative diagnosis of tubal cancer. The final pathologic diagnosis was pseudocarcinomatous hyperplasia of tubal epithelium associated with acute and chronic salpingitis in both tubes.

**Conclusion:**

The authors report a rare case of pseudocarcinomatous hyperplasia of the fallopian tubes mimicking tubal cancer.

## Background

Various benign conditions of the female genital tract may be confused with malignant neoplasms. Pseudocarcinomatous hyperplasia of fallopian tubes is a rare, reactive response to an underlying inflammatory or neoplastic process, and can mimic adenocarcinoma clinically and pathologically. Epithelial hyperplasia of a tube has been reported in association with estrogen administration, estrogenic ovarian lesions, tuberculous salpingitis, and nontuberculous salpingitis [1]. Mild to moderate epithelial stratification, nuclear atypia, and mitotic activity related to estrogenic stimulation might be observed in the tubal epithelium, but florid or atypical hyperplasia sufficient to be confused with adenocarcinoma is rarely seen.

Since this condition has not been discussed extensively in the literature, its differentiation from tubal cancer can be problematic [2], and morphologic similarities between pseudocarcinomatous hyperplasia and tubal cancer may be sufficient to cause significant misdiagnosis [3]. Differential features that aid the discrimination of benign pseudocarcinomatous hyperplasia of the fallopian tube and tubal cancer should be considered to ensure accurate diagnosis and proper management.

Herein, we present a case of pseudocarcinomatous hyperplasia of fallopian tubes with chronic salpingitis and endometriosis in tubes mimicking tubal cancer.

## Case presentation

**Fig. 1:**
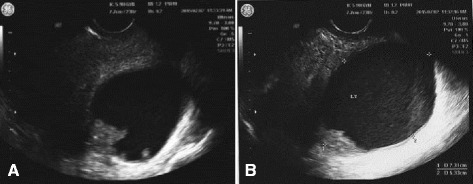
Transvaginal ultrasonography of the pelvis showing well-defined, bilateral, adnexal masses with papillary projections

**Fig. 2:**
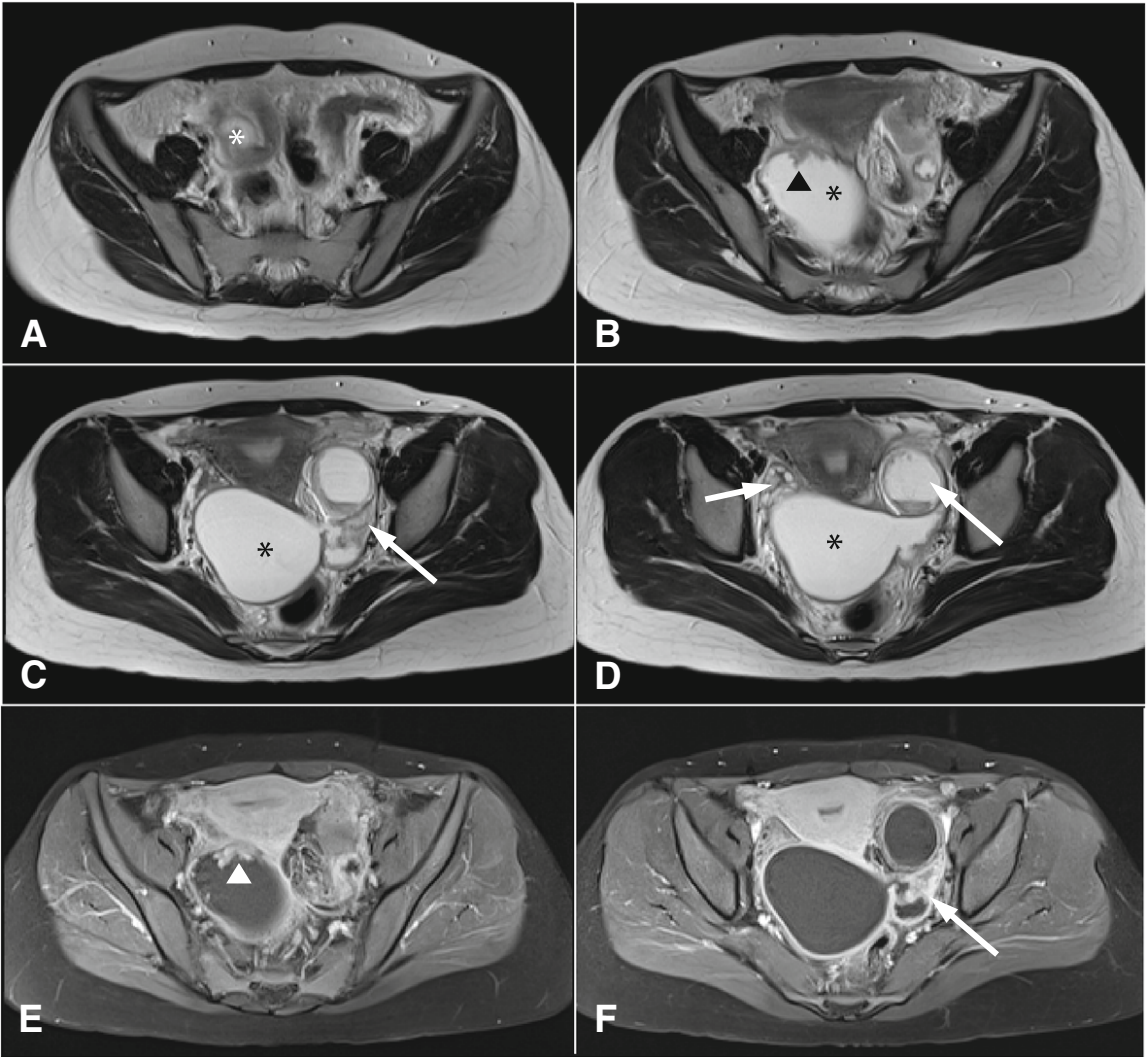
**a**-**d** Axial T2-weighted images showing bilateral adnexal complex cystic masses with fusiform or sausage-like shapes. The right adnexal mass (*) appeared as a cystic mass with papillary projections (*arrowhead*), whereas the left adnexal mass (long arrow) had an irregular thick wall. The right ovary (*short arrow*) was normal, but the normal left ovary was not visualized by MRI. (**e**-**f**) Contrast-enhanced fat-suppressed T1-weighted imaging revealed papillary projection enhancement (arrowhead) in the right adnexal mass and enhancement of the irregular thick wall (*long arrow*) in the left adnexal mass. These MRI features were suggestive of fallopian tube cancer

A 22-year-old nulliparous woman presented with persistent lower abdominal pain and vaginal spotting of 4 weeks’ duration. She was referred to our hospital due to bilateral adnexal masses and a highly elevated CA-125 level (1,056 U/ml). Pelvic ultrasonography showed well defined bilateral adnexal cysts with irregular thickened walls (Fig. 1). Magnetic resonance imaging (MRI) was performed to evaluate the pelvic mass further. Axial T2-weighted images showed bilateral adnexal complex cystic masses with fusiform or sausage-like shapes. The right adnexal mass appeared as a cystic mass with papillary projections and the left adnexal mass had an irregular thick wall. The right ovary was normal, but the left ovary was not visualized by MRI. Contrast-enhanced fat-suppressed T1-weighted images showed enhancement of papillary projections of the right adnexal mass and of the irregular thick wall of the left adnexal mass (Fig. 2).

**Fig. 3:**
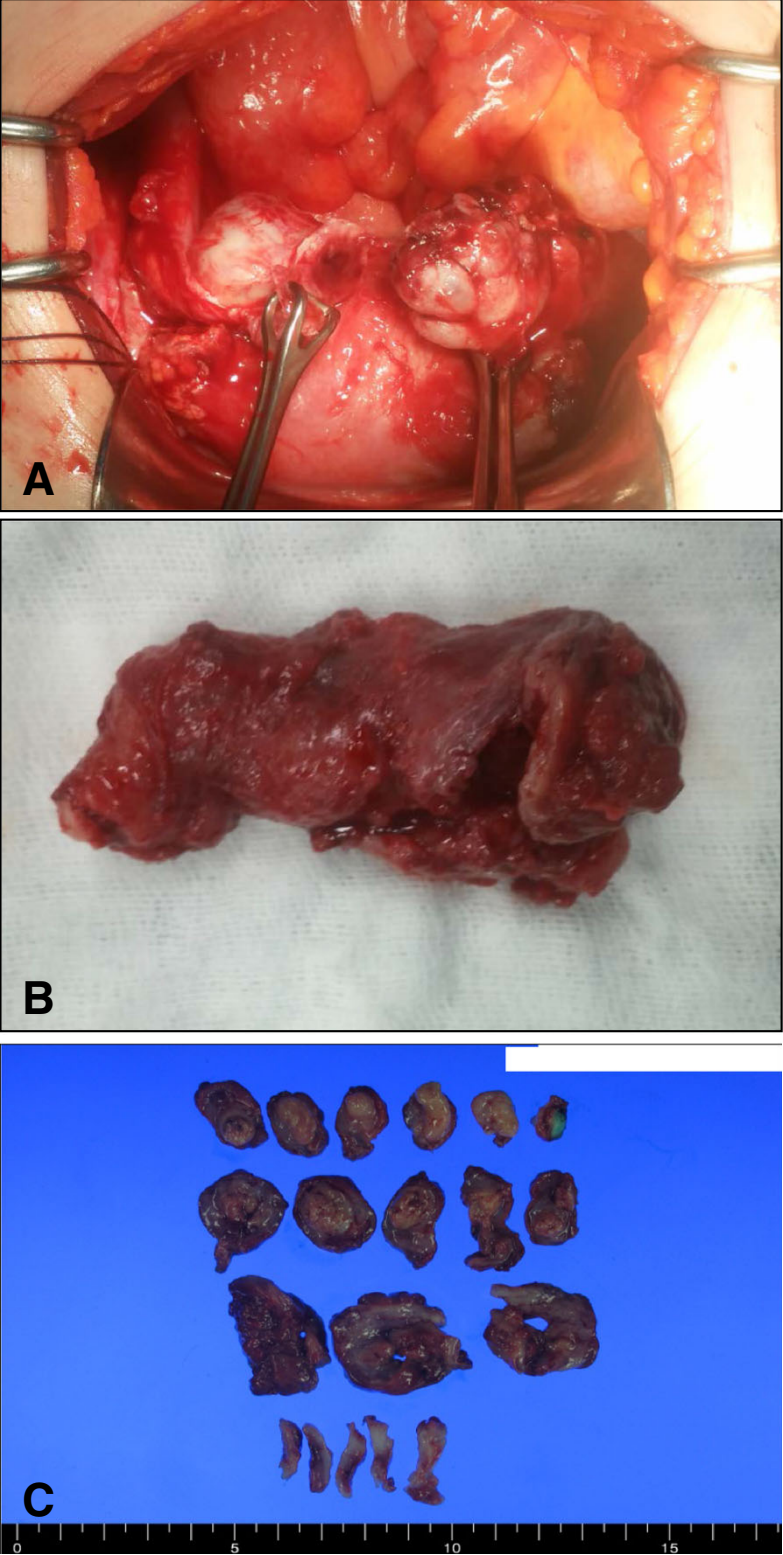
**a** Operative finding. Both tubes were resected and ovaries were grossly normal. The left ovary was partially resected and sutured due to adhesion and a ruptured surface (**b**) Gross appearance of the surgically resected tube. The tube was markedly dilated, thickened, and inflamed. **c** Multiple sections were taken for histopathological examination, but there was no gross evidence of tumor

Then, laparotomy was performed under a putative preoperative diagnosis of tubal cancer. Initially both ovaries were not visualized due to extensive firm, thick pelvic adhesions (Fig. 3a). Both tubes were enlarged with thickened walls. Intraoperative frozen section from right salpinx revealed acute and chronic inflammation but no evidence of malignancy. Bilateral salpingectomy was performed.

**Fig. 4:**
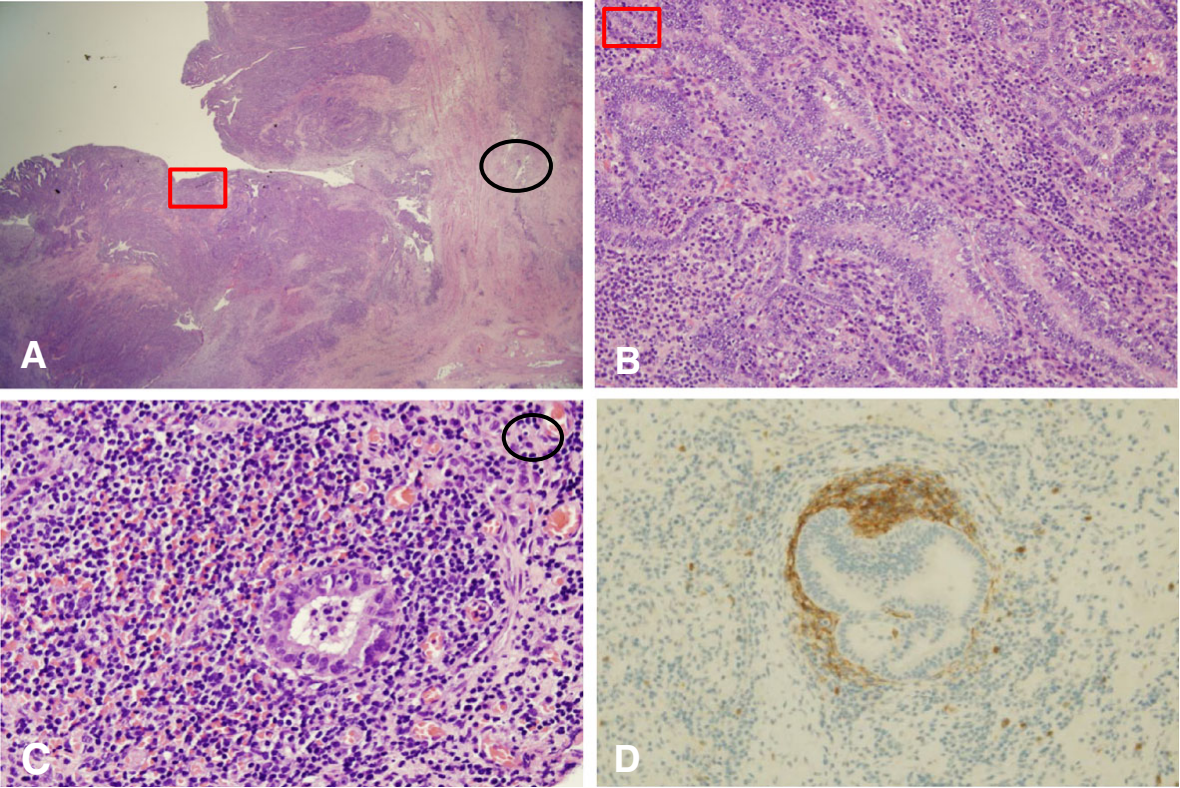
**a** Dilated fallopian tubes revealed marked mucosal hyperplasia under low power magnification (H&E, ×10). **b** Magnification of boxed area showing proliferating mucosa with nuclear crowding and epithelial stratification in a marked inflammatory background (H&E, ×200). **c-d** Magnification of the circular area showing foci of endometriosis on the outer fallopian tube wall (H&E, ×200 and CD10, ×200)

Grossly, both fallopian tubes were markedly dilated with thickened tubal walls (Fig. 3b). Microscopic findings revealed papillary growth and fusion of plicae (Fig. 4a). The tubal epithelium showed nuclear crowding and epithelial stratification with in a marked inflammatory background; however, cytologic atypia was minimal and mitoses were rare (Fig. 4b). Foci of endometriosis was observed on the outer walls of fallopian tubes (Fig. 4c), and CD10 immunostaining highlighted endometrial stroma at these foci (Fig. 4d).

The final pathologic diagnosis was pseudocarcinomatous hyperplasia of tubal epithelium associated with acute and chronic salpingitis in both tubes and endometriosis in the left tube.

Postoperative recovery was uneventful, and her CA-125 level normalized at 3 months after surgery. No recurrence occurred over 18 months of postoperative follow-up.

## Discussion

Pseudocarcinomatous hyperplasia is a pathologic diagnosis when a lesion exhibits florid epithelial hyperplasia with atypical features. However, pseudocarcinomatous hyperplasia of the fallopian tube is rare; a literature review revealed that ~14 cases have been issued on pseudocarcinomatous hyperplasia of the fallopian tube in patients aged 17 to 40 years [1]. About 50 % of these cases were associated with chronic salpingitis, whereas the others were associated with pyosalpinx, tubo-ovarian abscess, or hydrosalpinx (Table 1). Gupta et al. considered pseudocarcinomatous epithelial hyperplasia of fallopian tubes was related to female genital tract tuberculosis and reported that it histologically mimicked adenocarcinoma [2]. In our patient, it was associated with acute and chronic salpingitis, and endometriosis.

**Table 1:** Pseudocarcinomatous hyperplasia of Fallopian tubes

Authors	Age (years)	Cases	Clinical findings	Associated findings
Cheung et al.(1994) [1]	17-40	14	PID Tubo-ovarian mass	chronic salpingitis tubo-ovarian abscess pyosalpinx hydrosalpinx
Limaiem et al.(2000) [2]	32	1	secondary infertility	non-tuberculous chronic salpingitis
Gupta et al.(2012) [3]	35	1	persistent discharge dysmenorrhea oligomenorrhea	genital tract tuberculosis
Present case (2016)	22	1	lower abdominal pain vaginal spotting	acute and chronic salpingitis endometriosis of tube

Various benign conditions can be mistaken for malignant neoplasms, both clinically and pathologically. Microscopically, reactive atypical hyperplasia of fallopian tubes can be confused with carcinoma due to following microscopic findings: epithelial hyperplasia associated with a cribriform pattern, penetration of tubal wall by epithelium, or florid mesothelial hyperplasia. In initial experience, a radical hysterectomy was performed due to an erroneous diagnosis of carcinoma [1].

Pseudocarcinomatous hyperplasia is histologically differentiated from adenocarcinoma. Several morphologic features, such as, absence of a grossly detected tumor, presence of marked chronic inflammation, lack of solid epithelial proliferation, mild nuclear atypia, paucity of mitotic figures, and no evidence of invasion of the tubal wall, can help distinguish pseudocarcinomatous hyperplasia from tubal cancer, as in our case [1, 3] (Table 2). The paucity of mitotic figures has been considered as an important criterion to differentiate this lesion from carcinoma [2]. In our case, mitotic figures were absent. Pseudocarcinomatous hyperplasia can be differentiated from malignancy based on the morphologic features alone or combined use of morphologic features and additional immunohistochenical staining. The role of immunohistochemistry is quite variable between different cancer types. Unfortunately, immunohistochemical findings cannot aid the differentiation of pseudocarcinomatous hyperplasia of tubes and adenocarcinoma. Novak et al. suggest that Stathmin 1 (STMN1) and p16 are sensitive and specific adjunct biomarkers that, when used with p53 and Ki-67, improve the diagnostic accuracy of tubal carcinoma when compared to morphologically normal tubal epithelium [4]. These biomarkers might be helpful in difficult cases with diagnostic dilemma and the findings should be interpreted carefully.

**Table 2:** Clinico-pathological criteria for differentiation of pseudocarcinomatous hyperplasia of tubes from adenocarcinoma

Pseudocarcinomatous hyperplasia	Adenocarcinoma
1. Most patients are usually younger.	1. Most patients are postmenopausal, with a mean age of 62 years
2. It is always reactive and secondary. Usually associated with underlying chronic inflammation or hyperestrogenic states	2. It is always primary.
3. It shows no gross evidence of tumor, but there is inflamed, grossly dilated or thickened tube.	3. Most carcinomas are grossly evident.
4. Chronic inflammation is marked.	4. Chronic inflammation is not prominent.
5. Solid epithelial proliferation is not observed.	5. Solid epithelial proliferation is variably evident.
6. Mild to moderate nuclear atypia is observed	6. Nuclear atypia is prominent.
7. There are few mitotic figures. It has been considered an important criterion.	7. There are numerous mitotic figures.
8. Invasion of the tubal wall is not evident, but pseudoinvasion of the muscularis by gland like structures or lymphatic penetration by epithelial cells can be observed.	8. True invasion of the tubal wall is evident.

Several characteristics and features facilitate the differential diagnosis of pseudocarcinomatous hyperplasia and adenocarcinoma of the tube. First, patients with pseudocarcinomatous hyperplasia of tubes are younger. Second, there is bilateral diffuse involvement of tubes with no evidence of ovary involvement by any tumor. Third, patients with pseudocarcinomatous hyperplasia exhibit evidence of pelvic inflammatory disease (PID) either grossly or microscopically, whereas carcinomas do not.

No previous report has described the imaging appearance of pseudocarcinomatous hyperplasia of the fallopian tube. In our patient, it was visualized by pelvic ultrasonography and MRI as bilateral tubular cystic adnexal masses with papillary projections of the right mass and an irregular thick wall on the left mass. Furthermore, both of these features were intensely enhanced on contrast-enhanced MR images. The detection of a dilated fallopian tube by imaging aids determination that a cystic mass has a tubal origin, and is visualized as a thin-walled C- or S-shaped tubular cystic structure. The fluid is anechoic or shows low-level echoes by ultrasonography, and hyperintensity on T2-weighted images and hypointensity or hyperintensity (if hemorrhagic fluid is present) on T1-weighted images [5, 6]. In our case, enhancing nodular lesions and an irregular inner contour on MR images might have been caused by proliferative mucosal hyperplasia within fallopian tubes.

Radiologically, pseudocarcinomatous hyperplasia of fallopian tube may mimic tubal cancer, chronic salpingitis, or tubal tuberculosis. Tubal cancer also manifests as a solid, cystic adnexal mass, and the presence of enhancing intraluminal masses within a dilated tube is suggestive of its presence. On the other hand, thick-walled tubular adnexal cystic structures with intense enhancement and surrounding inflammation are indicative of salpingitis. However, thickened tube folding due to inflammation can be mistaken for enhancing mural nodules, and sometimes make it difficult to differentiate chronic salpingitis and tubal carcinoma [5, 6].

Pseudocarcinomatous hyperplasia of the fallopian tube is a rare disease and its radiologic features overlap with other fallopian tube diseases, such as, tubal cancer and salpingitis, and thus, intraoperative frozen section is needed to confirm the diagnosis.

Serum CA125 levels may provide information preoperatively that aid the differentiation of benign and malignant adnexal masses [7], and levels are significantly higher in patients with tube cancer than in patients with benign pelvic masses [3]. However, preoperative CA125 lacks the specificity needed to predict effectively the presence of malignancy [8].

## Conclusion

In conclusion, pseudocarcinomatous hyperplasia of the fallopian tube is a benign, reactive response to an underlying inflammatory process. Accurate discrimination is dependent on the identification of morphologic features, because there are no characteristic immunohistochemical findings that aid the differentiation of pseudocarcinomatous hyperplasia and tubal carcinoma. Thus, a meticulous morphological assessment is required to avoid an erroneous diagnosis of tubal cancer and subsequent overtreatment of this benign disease.

## Abbreviations

CA-125: Carbohydrate antigen-125
MRI: Magnetic resonance imaging
PID: Pelvic inflammatory disease
STMN1: Stathmin 1
